# Drug Therapeutic-Use Class Prediction and Repurposing Using Graph Convolutional Networks

**DOI:** 10.3390/pharmaceutics13111906

**Published:** 2021-11-10

**Authors:** Mapopa Chipofya, Hilal Tayara, Kil To Chong

**Affiliations:** 1 Department of Electronics and Information Engineering, Jeonbuk National University, Jeonju 54896, Korea; mapopachipofya@jbnu.ac.kr; 2 School of International Engineering and Science, Jeonbuk National University, Jeonju 54896, Korea; 3 Advanced Electronics and Information Research Center, Jeonbuk National University, Jeonju 54896, Korea

**Keywords:** medical subheading, drug function, drug repurposing, graph convolutional networks

## Abstract

An important stage in the process of discovering new drugs is when candidate molecules are tested of their efficacy. It is reported that testing drug efficacy empirically costs billions of dollars in the drug discovery pipeline. As a mechanism of expediting this process, researchers have resorted to using computational methods to predict the action of molecules in silico. Here, we present a way of predicting the therapeutic-use class of drugs from chemical structures only using graph convolutional networks. In comparison with existing methods which use fingerprints or images as training samples, our approach has yielded better results in all metrics under consideration. In particular, validation accuracy increased from 83–88% to 86–90% for single label tasks. Similarly, the model achieved an accuracy of over 88% on new test data. Finally, our multi-label classification model made new predictions which indicated that some of the drugs could have other therapeutic uses other than those indicated in the dataset. We performed a literature-based evaluation of these predictions and found evidence that validates them. This renders the model a potential tool to be used in search of drugs that are candidates for repurposing.

## 1. Introduction

Computational approaches are being widely explored in their use towards drug discovery, a process which has often been costly and time consuming [1,2,3]. One way to circumvent the arduous drug discovery process is to explore whether existing drugs could be used to treat other conditions other than those originally intended. This is known as drug repurposing. Drug repurposing reduces the cost to find new treatments for diseases, cuts short on time to do so, and has lower risk [3,4]. By using computational methods to identify the functions of drug compounds from chemical structures only, the drug repositioning and drug discovery process is accelerated further. This is the essence of this work.

Early work involving in silico methods in drug therapeutic-use class prediction strove to predict transcriptional response with functional properties of drugs [5,6,7]. The work presented in [8] attempted this by classifying drugs into therapeutic-use classes with deep neural networks solely based on transcriptional profiles. The limitation with these methods is that they require compounds to have associated transcriptomic measurements which may not be available. More recently, it was shown in [9] that chemical structures alone are effective predictors of therapeutic-use classes. In this work, the authors presented two methods. The first one involved training convolutional neural networks (CNN) using chemical images that represent the drugs. The images, which capture the drugs’ spatial chemical structure, were obtained using the open-source cheminformatics software RDKit [10]. This method was named IMG + CNN. The other method used Morgan fingerprints (MFPs) [11] to train random forests (RFs) [12] classifiers. This was called MFP + RF.

This work demonstrates the use of graph convolution networks in conjunction with graph representation of molecules based on their chemical structure to predict therapeutic-use classes. We chose to use graph-based representation of molecules because, by their very nature, drug molecules are graphs in which atoms are nodes and bonds correspond to edges. In addition, graph-based methods have provided good results in related areas such as predicting polypharmacy side effects [13], identifying drug repurposing opportunities [14], and in various areas of a molecular machine learning benchmark [15]. Although this work confirms that chemical structures alone are effective Medical SubHeading (MeSH) predictors, ostensibly it accentuates that, for this task, graph-based features and models are more effective than both images used together with CNN and molecular fingerprints used in conjunction with random forests. Our hypothesis is based on the fact that models trained on chemical images alone will not capture information beyond that provided in chemical images’ spatial structures. This was first observed in [16] where the authors noted that deep learning models’ performance improved by simply augmenting the same models with just three additional chemical properties which indicates that images alone might be incapable of capturing important chemical features. However, it is vital to note that the optimal way to represent chemical compounds to yield the best results using deep learning is still undetermined. Our more important contribution, however, is that our models make accurate predictions on test data and provide means for repurposing existing drugs. Here is how. We trained and validated our models on data, presented in [9], that were downloaded on 2 October 2018 and then tested them on new data that we downloaded from PubChem [17] on 28 January 2021. Only drugs that were not included in the initial dataset constituted the test set. Finally, our multi-label classification model has made new drug class predictions that were not available in test data but some of them have now been confirmed in literature. These confirmed predictions support the fact that the model is making meaningful predictions, thus making it an acceptable tool used for drug repurposing.

## 2. Materials and Methods

### 2.1. Overview of Our Approach

We have formulated the task to predict the MeSH class to which a drug belongs as a graph classification problem. More specifically, we have considered two main cases. Firstly, a case where each drug belongs to a single MeSH class only. This has been framed as a single label classification task. For this task, each drug has a single label in the entire dataset. In another case, a drug may belong to one or more MeSH classes. This has been framed as a multi-label classification task. The single label classification task is further subdivided into three task subgroups—3, 5, and 12. In the three-task subgroup, only drug compounds belonging to the central nervous system, antineoplastic, and cardiovascular MeSH classes were considered. In the five-task subgroup, gastrointestinal and anti-infective agents were included. Finally, in the 12-task subgroup, drugs belonging to all 12 MeSH classes were considered. A full list of the MeSH classes under consideration is provided in Table 1. The setup of these task subgroups is in line with what was conducted in [9] so that we can provide a comparison in performance of the models that is as fair as possible.

### 2.2. Data Preparation and Processing

#### 2.2.1. Acquiring Data

Drug therapeutic use classes can be found in the Medical Subject Headings (MeSH) thesaurus which is a controlled and hierarchically organized vocabulary produced by the National Library of Medicine. The PubChem substance and compound databases [17] website contains data classifying drugs into several MeSH classes. Data for training and validation purposes were obtained from the work presented in [9]. They downloaded this data from PubChem on 2 October 2018. We obtained the processed version of this data from their GitHub repository https://github.com/jgmeyerucsd/drug-class on 21 January 2021. There was a total of 6995 drug molecules that belonged to one MeSH class only. These were used for the single label classification tasks. A summary of the distribution of these is provided in the Training Samples column in Table 1. Another set containing 8336 drug molecules that belonged to one or more MeSH classes formed the set used for the multi-label classification task. The data for the test set was obtained directly from PubChem. To download the data, we followed the path: Browse data > Chemicals and Drugs Category > Chemical Actions and Uses > Pharmacologic Actions > Therapeutic Uses. We downloaded this set on 28 January 2021. Only drugs belonging to the 12 MeSH classes that appear in Table 1 were considered. The majority of the drug compounds in the new set were duplicates of those available in the training set. All such duplicates were removed from the new set. The remaining drug compounds comprised the test set. In the test set, a total of 1698 belonged to one MeSH class only. These were used to test the models trained for single label classification tasks. A summary of the distribution of this set is presented in the Test Samples column in Table 1. The value that is provided in parentheses in that column represents the proportion of the test drug compounds over the total number of drug compounds in that MeSH class, i.e., $\frac{x}{x+y}$ where *x* is the number of drugs in the test set and *y* is the number of drugs in the training set. Note that, for the majority of the MeSH classes, the test set represented a significant proportion of drugs on which to test the model. The respiratory system agents group presented the least proportion of new drugs—about 7% only. On the other hand, there were 4610 drug compounds that belonged to at least one MeSH class. These were used to test the model trained for the multi-label classification task.

#### 2.2.2. Converting SMILES into Graphs

**Figure 1:**
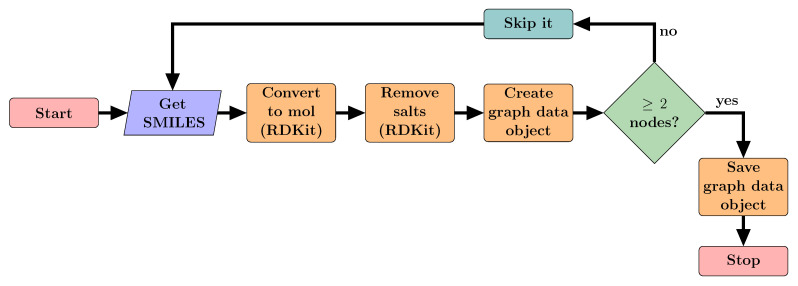
A flow chart showing how drug molecules were converted into and saved as graph data objects using RDKit [10] and PyG [18]. SMILES string representing drugs are converted into molecules using RDKit. Then salts are removed. The features from the atoms and bonds of the molecule are used to create a graph using PyG. This is then saved as a sample in the dataset.

Each of the drug molecule’s SMILES string was converted into an RDKit [10] molecule object. RDKit’s salt remover was then employed to remove salts, hence avoiding the mistake of keeping multiple versions of drug molecules that only differ because of the attached salts. From the resulting molecule object, we extracted the following information for each atom: atomic number, chirality, degree, formal charge, number of attached hydrogen atoms, number of radical electrons, kind of hybridization, whether it is aromatic, and whether the atom is in a ring. For bonds, we obtained the type, stereo, and whether a bond is conjugated or not. This information was then used to create a graph in which the nodes were atoms and the edges were the bonds. The node and edge features were, respectively, the atom and bond information described earlier. This way of encoding node and edge features is similar to that deployed in PyTorch Geometric, PyG [18], DeepChem [19], and GraphDTA [20]. PyG was used to create and save graph data objects. All graphs with fewer than two nodes were removed from the dataset as it would be difficult to use edge information from graphs with single nodes. Figure 1 shows a flow chart for this process.

### 2.3. Graph Convolution Networks

There are many learning methods on graphs, and libraries such as PyG [18] provide over 50 implementations of convolutional layers used in learning on graphs. Choice about which layers to use for a particular task is critical. In our case, we would ideally want to use a model that captures the information about the structure of the molecule’s graph and also have the capability to take higher-order graph structures at multiple scales into account. Such higher-order information has been shown to be useful in graph classification and regression tasks [15]. With that in mind, we decided to use the GraphConv layers provided in PyG that are an implementation of the higher-order graph neural networks put forward in [15]. GraphConv layers satisfy both of our requirements. Now consider the theory about GraphConv layers.

We can describe a graph *G* as a pair (V,E) where *V* is a finite set of nodes and E⊆{{u,v}⊆V|u≠v} is a set of the edges. Usually these sets are denoted V(G) and E(G), respectively. Similarly, the neighborhood of *v* in V(G) is denoted N(v). Often the set-based version of *k*-WL (from Weisfeiler–Leman algorithms) is used due to limited GPU memory. In that case, for a given *k*, *k*-element subsets ${\left[V\left(G\right)\right]}^{k}$ over V(G) are considered, resulting in the neighborhood $N\left(s\right)=\{t\in {\left[V\left(G\right)\right]}^{k}||s\cap t|=k-1\}$ where $s=\{s_{1},\ldots ,s_{k}\}$. For a labeled graph (G,l), in each *k*-GNN layer t≥0, the feature vector $f_{k}^{\left(t\right)}\left(s\right)$ for each *k*-set *s* in ${\left[V\left(G\right)\right]}^{k}$ is computed by

(1) $$f_{k}^{\left(t\right)}\left(s\right)=\sigma \left(f_{k}^{\left(t-1\right)}\left(s\right).W_{1}^{\left(t\right)}+\;\sum_{u\in N_{L}\left(s\right)}\;f_{k}^{\left(t-1\right)}\left(u\right).W_{2}^{\left(t\right)}\right)$$

where $W_{1}^{\left(t\right)}$ and $W_{2}^{\left(t\right)}$ are parametric matrices, σ represents a non-linear function. Moreover, $N_{L}\left(s\right)$ is the local neighborhood and consists of all t∈N(s), such that (v,w)∈E(G) for the unique v∈s\t and the the unique w∈t\s [15].

**Figure 2:**
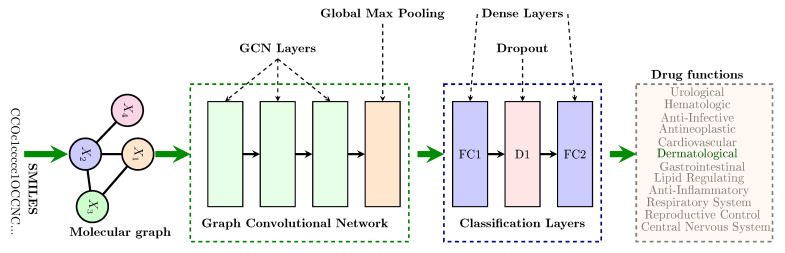
This figure depicts the architecture of the model used. The SMILES string representing each molecule is converted into a molecular graph. Then the graph convolution network learns a graph-based embedding for that molecule. This is then passed to the subsequent fully connected (dense) layers for classification. FC2 has 3, 5, or 12 outputs commensurate with the classification task.

Figure 2 shows the architecture of our basic model. As illustrated in that figure, SMILES (Simplified Molecular Input Line Entry System) [21] strings representing drugs are converted into molecular graphs. These graphs are then fed to the model to learn a latent representation vector for each drug in the graph convolution network block. In that block, each GCN layer subblock comprises a GraphConv layer, a Rectified Linear Unit (ReLU) activation function, and a GraphNorm layer, respectively. The latent vector, chosen to be of length 1024 after some experimentation, is subsequently pushed through the classification layers to predict the class to which a drug belongs. We used the log softmax activation at the output of FC layer 2 and applied the negative log likelihood loss for classification in the single label tasks. In the multi-label classification tasks, the log softmax layer was not included and a multi-label soft margin loss was applied. All these activation and loss functions are implemented in PyTorch [22].

Best parameters for the models were obtained by using the Optuna [23] hyperparameter optimization library. These parameters were then used in building PyTorch models. Table 2 provides information about important hyperparameter settings we used in our models.

### 2.4. Training Details

For single label tasks, 5-fold stratified cross validation was used, whereas for the multi-label classification task, data was split into five folds based on pairwise co-occurrence using the iterative class splitter from the skmultilearn [24] package. The exact folds used in [9] cross validating IMG + CNN and MFP + RF models were also employed in this experiment.

MeSH classes were assigned weights to take into account the effects of the imbalanced nature of the data during training. For a given MeSH class *i*, the normalized weight $w_{i}$ assigned to each MeSH class was defined as

(2) $$w_{i}={\left(c_{i}\sum_{k=1}^{N}\frac{1}{c_{k}}\right)}^{-1}$$

where $c_{i}$ and $c_{k}$ are the total number of drugs belonging to MeSH classes *i* and *k*, respectively, and *N* is the number of MeSH classes considered for that task subgroup (3, 5, or 12). The vector containing these weights for each task subgroup was then provided to the PyTorch loss function as an argument.

### 2.5. Performance Evaluation Metrics

The performance for each of the models was assessed using several statistical metrics. For single label classification, tasks metrics such as accuracy, balanced accuracy (BAC)—which helps to circumvent the problem of inflated performance estimates on imbalanced datasets such as the one used here—Matthew’s correlation coefficient (MCC), Area Under the Receiver Operating Characteristic Curve (AUROC), and average precision (AP) were employed. These are defined mathematically as

(3) $$\mathrm{Accuracy}=\frac{TP+TN}{TP+TN+FP+FN}$$

(4) $$\mathrm{BAC}=\frac{1}{2}\left(\frac{TP}{TP+FN}+\frac{TN}{TN+FP}\right)$$

(5) $$\mathrm{MCC}=\frac{TP\times TN-FP\times FN}{\sqrt{\left(TP+FP)(TP+FN)(TN+FP)(TN+FN\right)}}$$

Average precision (AP) is dependent on precision, PRE, and recall, REC, where

$$PRE=\frac{TP}{TP+FP}$$

$$REC=\frac{TP}{TP+FN}$$

and TP, true positive, denotes the number of drug compounds that are correctly predicted to belong to each MeSH class; TN, true negative, represents the number of drugs that are correctly predicted not to belong to each MeSH class; FP, false positive, is the number representing those drugs that are incorrectly predicted to belong to each MeSH class but do not actually belong to that class; and FN, false negative, is the number of drugs that belong to one MeSH class but have been incorrectly predicted to belong to other classes. If $PRE_{n}$ and $REC_{n}$ represent the precision and recall at the nth threshold, we can obtain the average precision by

(6) $$\mathrm{AP}=\sum_{n}^{}\left(REC_{n}-REC_{n-1}\right)PRE_{n}$$

For the multi-label classification task subgroup, we employed accuracy, AUROC, and AP, as in the single label tasks, in addition to the Fbeta score ($F_{\beta }$). The Fbeta score is an abstraction of the F-measure where the balance of precision and recall in the calculation of the harmonic mean is controlled by a coefficient called β.

(7) $$F_{\beta }=\frac{(1+\beta^{2})PRE\times REC}{\beta^{2}\times PRE+REC}$$

where *P* is precision and *R* is recall, defined previously.

### 2.6. Model and Representation Comparisons

Comparison of the models’ performance on cross validation and test data using the metrics provided in Section 2.5 was performed as follows. The mean and standard deviation for each metric across the five folds was recorded, and these are presented in the tables of results. However, for the confusion matrices, performance of all models was compared on one representative validation fold—the fifth fold. Furthermore, figures showing distributions of the data after modeling, and predicted networks of drugs, are those obtained using the model trained on this representative fold.

For each task subgroup, we sought a way of showing the distribution of the drug compounds for that task, before and after modeling. Node and edge features of each graph representing a drug compound are high-dimensional tensors. As such, they cannot be directly plotted onto a two-dimensional (2D) space. To reduce the dimensions of the data to 2D, Uniform Manifold Approximation and Projection (UMAP) [25] was used. Each plot of data distribution before modeling was obtained by passing it through a dummy GCN model with weights initialized to all ones. The output was then fed to UMAP to obtain a 2D vector for each drug. On the other hand, the distribution of the data after modeling was obtained by passing each drug compound through the model for that particular task subgroup and using the latent vector attained at hidden layer FC1, shown in Figure 2. This latent vector was then fed to UMAP to obtain a 2D vector for each drug. All plots related to UMAP plotted using Seaborn statistical data visualization tool [26].

For the multi-label classification task, we employed networks of relationships in the analysis of results. Networks are ubiquitous and can quickly provide information about the nature and degree of interactions between items and enable inferences about the reason for those interactions [27]. Inherently, the multi-label classification task renders itself easy to analysis using networks. All figures showing network relationships were drawn using NetworkX [28] and Pyvis [29].

## 3. Results

In this section, we present results of our models for all the four tasks and compare our models with those rendered in [9]. We first consider results of our single label classification models on cross validation in Section 3.1. Then we present results of our single label classification models, but this time on test data, in Section 3.2. Thereafter, we consider results of our multi-label classification model in comparison with previous models in Section 3.3. Lastly, we consider drug repurposing opportunities as predicted by our models in Section 3.4.

### 3.1. Single Label Classification Results for Cross Validation

Table 3 provides a summary of validation and test results for the three single label tasks. If we consider the results on cross validation from that table, it is observed that the average metrics of our GCN based models are higher compared with the other models—IMG + CNN and MFP + RF. GCN based models outperformed the other models in all categories and in all metrics considered, except on average precision score for the three-task subgroup. The good results obtained from GCN models could be attributed to the fact that the information about the molecules’ bonds and atoms is well captured and learned in graph-based methods than perhaps from images or fingerprints as accentuated in [15].

**Figure 3:**
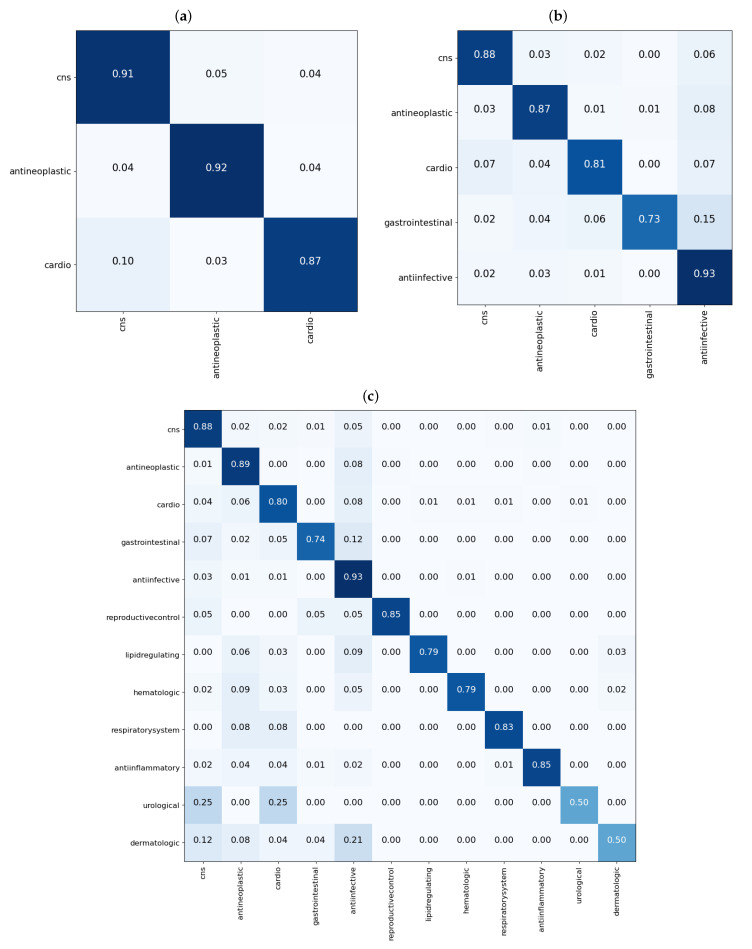
Confusion matrices showing performance of the GCN models on the fifth fold of the validation data for the single label tasks. In (**a**), results for the 3-task subgroup; in (**b**), results for the 5-task subgroup; and in (**c**), results for the 12-task subgroup. Each matrix shows the proportion of each predicted class, on the x-axis, for molecules in each true class on the y-axis.

**Figure 4:**
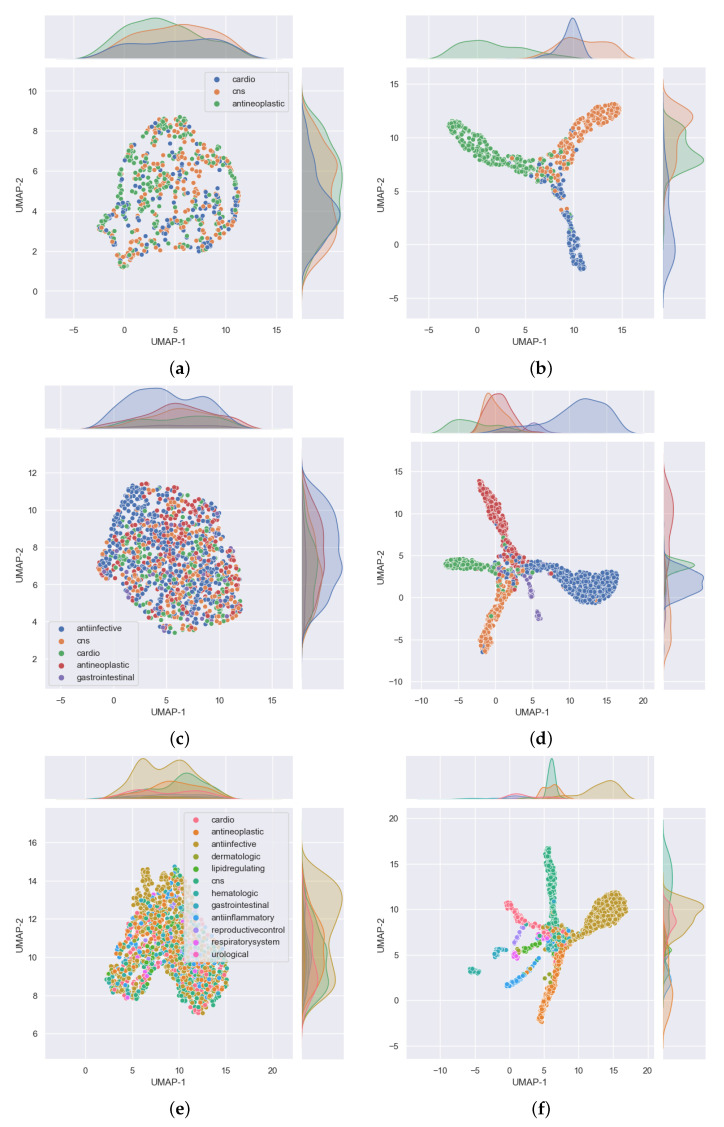
Distribution of the fifth fold cross validation data used in the 3, 5, and 12 single label classification task subgroups shown on (**a**,**c**,**e**), respectively. On the other hand, (**b**,**d**,**f**) show the distribution of the same data after passing them through our trained GCN models for 3-, 5-, and 12-task subgroups, respectively. The latter group of subfigures show that drugs belonging to one MeSH class appear closer to each other in the 2D UMAP space (which is not the case in the earlier group of subfigures). This shows that the models are able to classify the drugs into the 3, 5, and 12 MeSH classes as intended. These subfigures were plotted using Seaborn [26] after we reduced the dimensions of the drugs to two using UMAP [25].

From the results shown in the confusion matrices of Figure 3, we can see that our models yield good results on cross validation. In the three-task subgroup (Figure 3a), antineoplastic agents were predicted more accurately (92%) followed by central nervous system agents (91%) and then cardiovascular agents at 87%. Cardiovascular agents were the least well predicted, probably because they were the smallest set in this task with only 788 compounds compared with 1177 for antineoplastic and 1139 for central nervous system agents. The disparity in the numbers can further be observed in Figure 4. In that figure, Figure 4a shows the distribution of the data that was used for validation. It can be seen that, for the most part, the drugs in each class are evenly distributed, but the data are imbalanced among the three classes. There are many more antineoplastic and central nervous system agents as there are cardiovascular agents. Similar disparities will become more apparent in the 5- and 12-task subgroups. However, it is important to note that, after passing this data into our model, the model clearly classifies the drugs into three classes with an accuracy of over 90%, as shown in Table 3 (under the GCN Val column). The good performance of the model is further evidenced in Figure 4b, where we can see drugs belonging to each of the three MeSH classes clustered closer together as determined by the model.

In the five-task subgroup, whose results are summarized in the confusion matrix of Figure 3b, anti-infective agents were predicted more accurately (93%). A total of 2398 compounds belonging to this group were used for cross validation (Table 1). In contrast, there was a total of 258 drug compounds belonging to the gastrointestinal group for this task. Gastrointestinal agents were the least well predicted, with an accuracy of 73%. As with the three-task subgroup, central nervous system agents and antineoplastic agents, which are relatively equal in numbers, yielded similar accuracies of 88 and 87%, respectively. Moreover, as in the three-task subgroup, the cardiovascular group achieved an accuracy of 81% which is lower than that of either the CNS or antineoplastic groups. Again, there are some disparities in the numbers of drugs belonging to each class that have been used for this task, as observed in Figure 4. In that figure, the distribution of the data that were used for validation is presented in Figure 4c. As before, despite the data being imbalanced, the model clearly classifies the drugs into the five MeSH classes, with an accuracy of over 88%, as shown in Table 3 (under the GCN Val column). The good performance of the model is further evidenced in Figure 4d, where it is clear to see drugs belonging to each of the five MeSH classes distributed closer to each other after modeling.

Much like in the five-task subgroup, results of the 12-task subgroup presented in Figure 3c indicate that the 2398 anti-infective agents were predicted more accurately (93%). In contrast, the set of 26 urological agents only yielded an accuracy of 50%, presumably because they were very few. Perhaps the most difficult group to predict was the dermatological agents. With a total number of 115 compounds belonging to this group, only 50% of those used for validation in the fifth fold were correctly predicted. In addition, 21% (≈5) of drugs used for validation in the fifth fold were wrongly predicted as belonging to anti-infective agents. Reminiscent of the previous two task subgroups, the disparity in the numbers of drugs belonging to each class are more prominent in the 12-task subgroup, evident in Figure 4e,f where Figure 4e shows the distribution of the data. It can be seen that, for the most part, the drugs in each class are evenly distributed and the data is highly imbalanced. As noted earlier, urological agents are much less compared with the other classes. However, it is important to note that, after passing this data into our model, the model is able to classify the drugs into the 12 MeSH classes with an accuracy of over 86%, as shown in Table 3 under the GCN Val column. The good performance of the model is further evidenced in Figure 4f, where we can see 12 clusters representing the 12 MeSH classes emerging after modeling.

### 3.2. Single Label Classification Results on Test Data

This section provides results showing the performance of our single label classification models on the test dataset.

**Figure 5:**
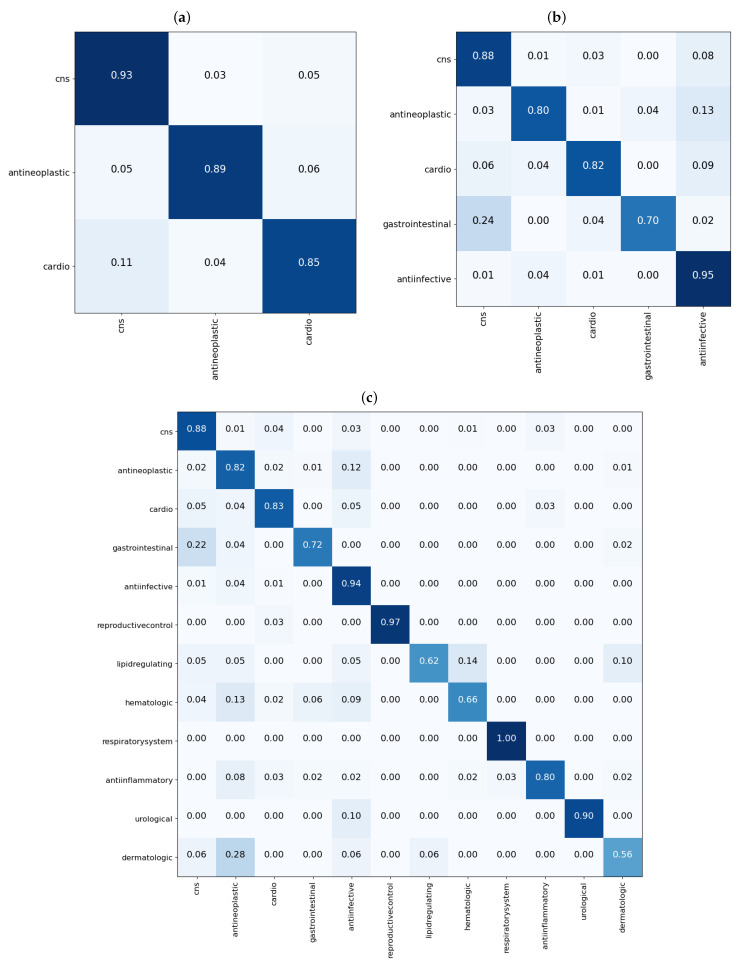
Confusion matrices showing performance of the GCN models on the test data for the single label tasks. In (**a**), results for the 3-task subgroup; in (**b**), results for the 5-task subgroup; and in (**c**), results for the 12-task subgroup. Each matrix shows the proportion of each predicted class, on the x-axis, for molecules in each true class on the y-axis.

From the results shown in the confusion matrices of Figure 5, we can see that our models yield good results on the test data as well. Results of the three-task subgroup, which are presented in Figure 5a, indicate that central nervous system agents were predicted more correctly (93%) than the other two classes. Akin to the cross validation results, cardiovascular agents were the least well predicted (85%). Overall, the performance of the model on test data is comparable to that obtained from cross validation in this task subgroup. As an example, the test accuracy for this task is about 88%, as shown in Table 3 (under the GCN Test column), compared to a validation accuracy of 90% (under the GCN Val column).

**Figure 6:**
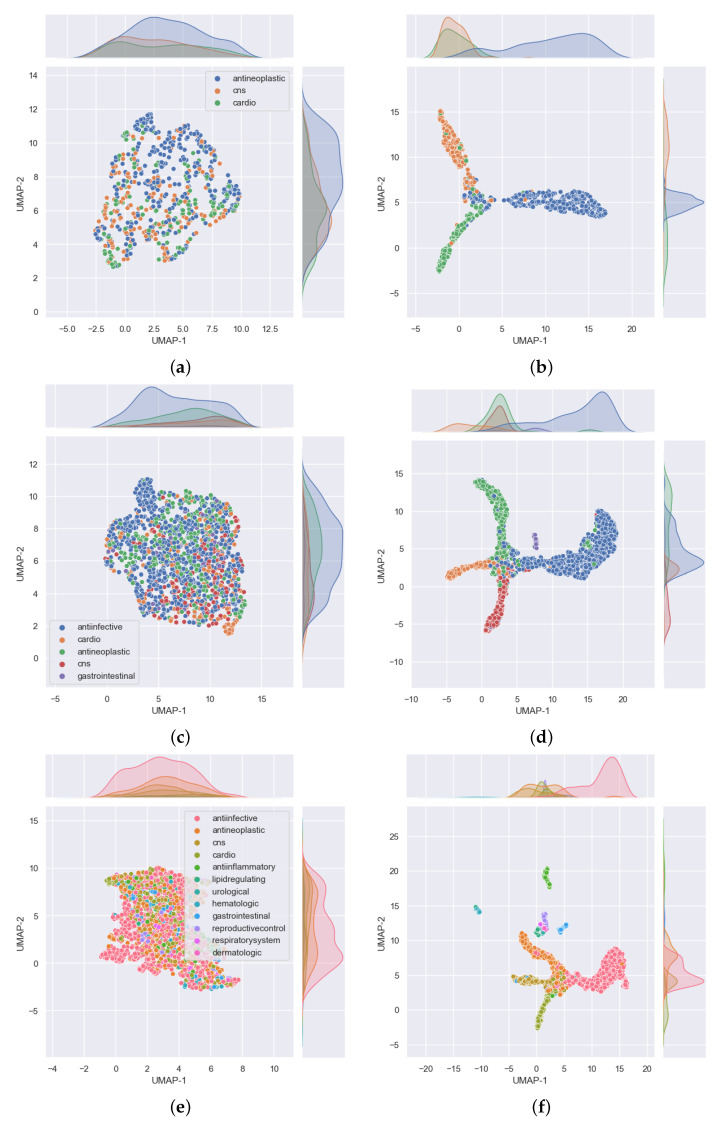
Distribution of the test data used in the 3, 5, and 12 single label classification task subgroups shown on (**a**,**c**,**e**), respectively. On the other hand, (**b**,**d**,**f**) show the distribution of the same data after passing it through our trained GCN models for 3-, 5-, and 12-task subgroups, respectively. The latter group of subfigures show that drugs belonging to one MeSH class appear closer to each other in the 2D UMAP space (which is not the case in the earlier group of subfigures). This shows that the models are able to classify the drugs into the 3, 5, and 12 MeSH classes as intended. These subfigures were plotted using Seaborn [26] after we reduced the dimensions of the drugs to two using UMAP [25].

Figure 6a shows the distribution of the data that was used for testing in the three-task subgroup. It is observed that the drugs in each class are fairly evenly distributed on both axes without any conspicuous outliers, but there are many more antineoplastic agents (342) as there are cardiovascular (152) and central nervous system (176) agents. After passing this data into our model, the model classifies the drugs into the three MeSH classes and the majority of these appear closer to each other in the 2D space, as shown in Figure 6b.

Performance results on test data for the 5-task subgroup are analogous with those obtained from cross validation. From Table 3, under the GCN Val and GCN Test columns, the accuracy in both cases is about 88%. Moreover, checking on the confusion matrices of Figure 3b and Figure 5b, it is observed that 88% of CNS agents were correctly predicted in both cases, cardiovascular agents achieved 81 and 82%, respectively, whereas anti-infective agents registered 93 and 95%. Antineoplastic agents achieved 87 and 80%, in that order, while gastrointestinal agents recorded 73 and 70%.

Figure 6c reveals that the distribution of the data used for testing was evenly dispersed, but there were big disparities in the number of drugs among the classes. In particular, there were about 776 anti-infective agents in contrast to 46 gastrointestinal agents. This disparity is also well depicted in Figure 6d, which shows five noticeable MeSH classes in which drugs belonging to each class appear closer to each other after the model correctly classified the majority of the drugs into their respective classes.

In the 12-task subgroup, the respiratory system agents class was perfectly predicted at 100%, as shown in the confusion matrix of Figure 5c. This was also the smallest group in the test data with just eight drug compounds. The urological agents were the overall smallest dataset, with 10 drugs in the test set and 26 drug compounds used in cross validation (Table 1). However, the model correctly predicted 90% of the test drugs in this class (Figure 5c).

The overall accuracy on test data for this task is comparable to that obtained from cross validation. From Table 3, the accuracy in either case is about 86%. Figure 6e shows the distribution of the data that was used for testing. It is clear that there are many more anti-infective agents (776) than the other classes. However, the drug compounds are fairly evenly distributed along both axes. Results of distribution of the same data after passing it through our model are provided in Figure 6f, where the majority of drugs belonging to each MeSH class are in close proximity in the 2D space as evidence of the model’s ability to classify them into the 12 MeSH classes.

### 3.3. Multi-Label Classification Results on Validation and Test Data 

In this section, we explore the capacity of the model to predict whether a drug belongs to more therapeutic-use classes. This provides us with an opportunity to learn from the undetermined predictions that these models make so that we can act accordingly in case some drugs could be fit for repurposing.

A total of 8336 molecules were present in one or more MeSH classes compared with 6995 molecules which belonged to a single MeSH therapeutic-use class only. This represents an average of 1.2 MeSH class labels for each molecule. On the other hand, the test dataset contained 4610 drug molecules belonging to one or more MeSH classes and 1698 molecules which belonged to one MeSH class only, representing an average of 2.7 MeSH class labels for each molecule.

Metrics’ results for multi-label classification on validation and test data are presented in Table 4. Here, too, our model performs better than IMG + CNN. In particular, the $F_{\beta }$ score, which was around 64% when using IMG + CNN, rose to over 79% when our GCN base model was used.

**Figure 7:**
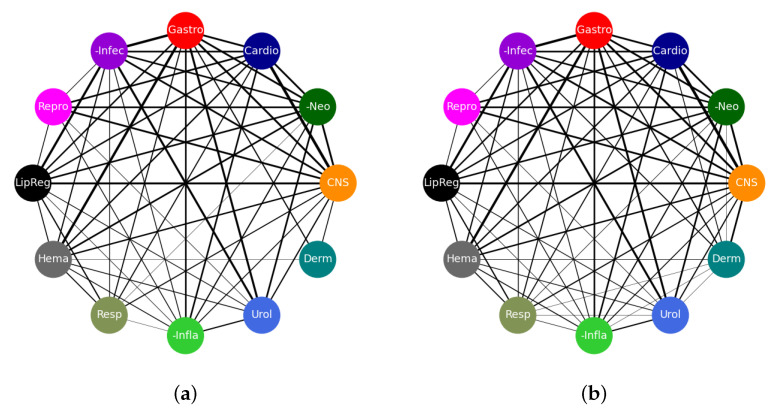
Networks of multi-label classification on the test dataset. The connections as given by true class labels are shown in (**a**) while those predicted by the model are shown in (**b**). In each of these subfigures, the thicker the edge the higher the frequency of co-occurrence of nodes. There are seven edge types that appear in the network of predictions that do not appear in the network of true class labels. These are urological–dermatological, antineoplastic–dermatological, respiratory–urological, anti-inflammatory–dermatological, anti-infective–dermatological, cardiovascular–dermatological, and respiratory–dermatological. Such new edge types show the obvious misclassifications made by the model. Other misclassifications are more subtle and can be seen by the widths of the edges. We have provided the list of edge counts for that case in the Supplementary Material. Networks in this figure were drawn using NetworkX [28].

These good results are also reflected in the connectivity diagrams shown in Figure 7. In these diagrams, a single edge represents a drug that belongs to the two classes to which that edge is attached. Thicker edges represent more drugs on that edge and, conversely, thinner edges represent fewer drugs that belong to the two connected classes. No connection between nodes means that there are no drugs that belong to both concerned nodes. The width, *w*, of each edge was computed using $w=\frac{1}{2}\log_{2}\left(N+1\right)$ where *N* is the number of co-occurrences of nodes on each edge. The network of Figure 7a represents the true connections as dictated by the true class labels while the one in Figure 7b shows a network of predictions by our model. In total, there are 4610 edges in each of the networks. A total of 4210 edges are exactly the same while 400 edges appear in one network but not the other. This shows that our model has made a majority of the predictions accurately which is in line with the results in Table 4. The edges that appear in Figure 7a, but not in Figure 7b, have been thought of as those the model has failed to learn, whereas those that appear in Figure 7b only have been considered to be areas where the model may have learnt something new and we need to verify if that is the case. This is also one area where drug candidates for repurposing could be found.

### 3.4. Drug Repurposing Opportunities

While it is paramount to make sure the model classifies the drugs accurately, it is also important to pay particular attention to cases where the model makes some misclassifications. In general, we tend to consider such misclassifications as an indication that the model has not learnt enough about the data. However, in some cases, it could be an indication that the model has learnt something new about the data. Such cases require that we verify the model’s results using alternative approaches. In this situation, we are going to check the literature for evidence of the predictions that the model has made but do not tally with those given in the dataset.

**Figure 8:**
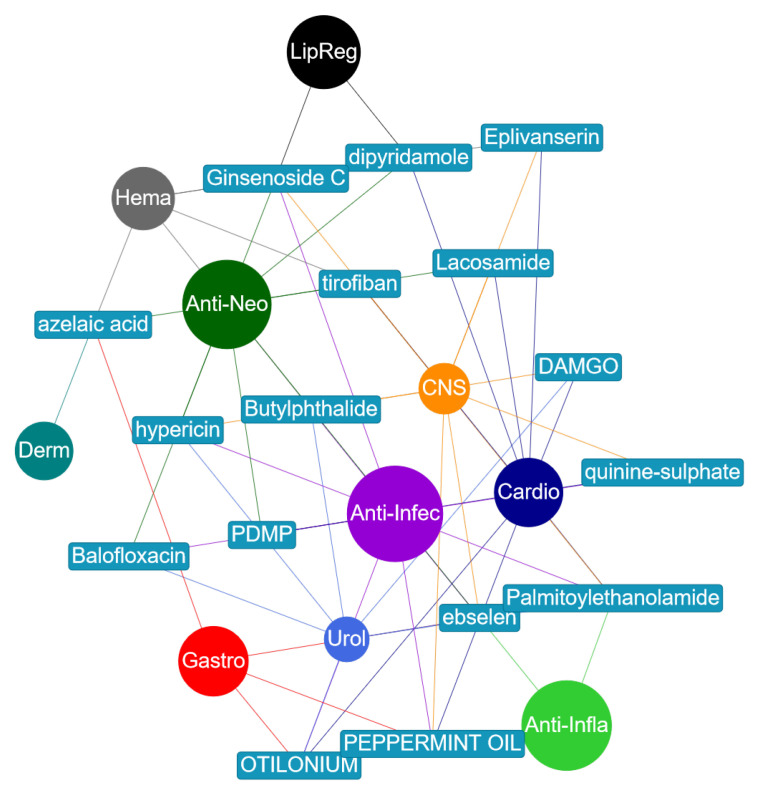
A network of misclassified drugs. The MeSH classes are presented as circular nodes whereas drugs are shown in rectangular boxes. Each drug is connected to two or more MeSH classes to which it has been predicted to belong. An edge linking a drug node and a MeSH class node inherits the color of the MeSH class node. Thus, drug nodes have different colors for edges attached to them whilst MeSH class nodes have the same color to all their edges. This network was drawn using Pyvis [29].

Figure 8 shows a small network of the drugs that were misclassified but have evidence in literature backing those predictions to be true. This information is also provided in Table 5 where citations to literature that provides the evidence have been made.

For example, ginsenoside Rb2 was given as an antineoplastic and a lipid regulating agent. However, the model predicted that it was an anti-infective and a CNS agent. This compound, also known as ginsenoside C, is a ginsenoside found in *Panax ginseng*. It is indicted on the Chemical Entities of Biological Interest (ChEBI) [31] website, stub CHEBI:77152, that the compound has a role as an antiviral agent which confirms that it is an anti-infective agent. As regards the compound being a CNS agent, it was reported in [32] that ginsenoside Rb2 greatly activated Cu,Zn-superoxide dismutase gene (SOD1) through transcription factor AP2 binding sites. SOD1 is one of the major antioxidant enzymes and its presence significantly delayed the onset of signs of motor impairment and prolonged the survival of mice suffering from Amyotrophic Lateral Sclerosis (ALS). ALS is a progressive neurodegenerative disease characterized by degeneration of motoneurons in the spinal anterior horn.

Similarly, Balofloxacin was given as an antineoplastic and anti-infective agent. The model predicted that it is also a urological agent. In [34], Balofloxacin was found to be effective in 78.9 and 82.4% of patients with complicated urinary tract infection (UTI) assessed by the physician’s evaluations and Japanese UTI criteria, respectively.

Dipyridamole (DIP) was given as a cardiovascular and hematological agent. In a reverse screening approach to identify potential anti-cancer (antineoplastic) targets of DIP, it was noted in [35] that DIP can increase the anti-cancer drug (5-fluorouracil, methotrexate, piperidine, vincristine) concentration in cancer cells and hence enhance the efficacy of treatment of cancer. This is in line with the model’s prediction of DIP being a potential antineoplastic agent. Furthermore, there are reported (in vitro and in vivo) results in [36] which demonstrate that the suppression of HMGCS1 (3-hydroxy-3-methylglutaryl-CoA synthase 1) by siRNA and dipyridamole enhances the antitumor properties of trametinib in colon cancer cells. The work presented in [37] reported that DIP inhibits lipogenic gene expression by retaining SCAP-SREBP in the endoplasmic reticulum. This is consistent with both predictions about DIP being a prospective antineoplastic and lipid regulating agent.

Likewise, hypericin was given as an anti-infective, antineoplastic, and CNS agent. The model predicted that it is also urological. Reports in [38,39,40] indicate that it has been used in urological medicine as a photo diagnostic to detect non-muscle-invasive bladder cancer lesions.

Lacosamide was given as a cardiovascular and a CNS agent. The model predicted that it is also antineoplastic. This was confirmed in [41], where results of in vitro antineoplastic effects of brivaracetam and lacosamide on human glioma cells were reported. It was further reported in [42,43] that lacosamide, when added to any baseline anti-epileptic drug, is effective in obtaining a high seizure reduction and seizure freedom, regardless of the tumor activity and response to antineoplastic therapies.

Further, otilonium bromide was provided as a cardiovascular and a gastrointestinal agent. However, the model predicted that it is an anti-infective and a urological agent. Recent results presented in [44] indicate the drug could be a new antimicrobial agent to treat *Staphylococcus aureus* infections more safely and efficiently. Information about it being urological has not been found yet.

Palmitoylethanolamide (PEA) was given as an anti-infective, an anti-inflammatory, and a CNS agent. The model predicted it as also being a cardiovascular agent. It was reported in [45] that PEA evokes potent anti-inflammatory effects in cultured macrophages which translates under in vivo conditions into a strong atheroprotective effect. In addition, based on the clinical studies for testing PEA to treat pain and inflammation that were going on at the time, PEA was thought to be an interesting novel therapeutic drug for patients with cardiovascular disease. Similarly, research results reported in [46] indicate that PEA combined to polydatin protects against cardio and vasculotoxicity of doxorubicin by promoting an anti-inflammatory phenotype, representing a new therapeutic approach to resolve doxorubicin-induced cardiotoxicity and inflammation. We have not yet found information that confirms whether PEA could be a urological agent.

Peppermint oil was given as a CNS and a gastrointestinal agent. Results from the work presented in [47] indicated the strong antibacterial and antioxidant activities of peppermint oil. This is in line with the model’s prediction that the compound is a potential anti-infective agent. In a recent study [48], it was shown that aromatherapy with peppermint essential oils can improve the sleep quality of cardiac patients and was recommended for cardiac patients as a non-pharmacological intervention. This agrees with the model’s prediction of it being a cardiovascular agent.

Finally, tirofiban was given as a cardiovascular and a hematologic agent. The model predicted it as a CNS and an antineoplastic agent. Tirofiban has been reported to constrain tumor cell invasive potential in HSC-3 human tongue squamous cell line [49,50]. It has also been reported that low-dose tirofiban treatment improves neurological deterioration outcome after intravenous thrombolysis [51]. This is in line with the model’s second prediction that this compound could also be a CNS agent.

## 4. Discussion

We have demonstrated here that going a step further to scrutinize the predictions the models make is one of the key facets that might lead to finding more drugs that are candidates for repurposing. This process can also be improved by enhancing the interpretability of deep learning models in such a way that we can learn directly about the substructures (atoms and bonds) within the drug’s molecular structure that contribute more to the model’s prediction about that drug. Knowledge about such substructures could also help in the design of new drugs. Currently, the Captum [52] library for model interpretability built on PyTorch can help in that aspect. Consider also that, in this experiment, we only looked at drugs that belong to twelve MeSH classes. On the PubChem website there are over twenty MeSH classes and, as such, models built to classify drugs for all those MeSH classes would be more helpful. In addition, APIs or web tools for easy access to such models would be a very welcome development. Notice also that we did not check the misclassifications for single label tasks. The rationale behind not checking those is that we used 6995 drug molecules for training single label classification models and 8336 molecules for training the multi-label classification model, and so the multi-label model learned to generalize better and hence could be trusted more because it was trained on more samples. Moreover, all drugs that were used in training the single label classification task were also part of the 8336 drugs used in the multi-label classification. However, it is ideal to check the misclassifications made by both models. The use of both single label and multi-label classification models in this manner could potentially act as a quick way to narrow down on candidates for repurposing. For example, if drug A is misclassified by both models as belonging to MeSH class X then that drug should be prioritized when checking the literature.

## 5. Conclusions

In this work, we have presented a method for predicting therapeutic-use classes of drugs from the features of their molecular graphs which are dependent on their corresponding chemical structures. We have demonstrated that our approach performs better than existing methods that also used chemical-derived features but did not contain graph-related information of the drug compounds. The great improvement demonstrated by our approach underscores the importance of incorporating graph information in artificial intelligence models that are applied to chemical structures which are graph in nature. More importantly, we have demonstrated that our model has the capability to make accurate predictions on unseen data. We achieved this by testing our model on data that appeared on a later date than the data on which it was trained. We have further presented results of new predictions that indicate repurposing opportunities, some of which have been confirmed by evidence in literature.

## Figures and Tables

**Table 1:** Summary of the distribution of 6995 training samples and 1698 test samples. The test samples consisted of drug compounds that were not present in the training samples. The classification tasks to which drug compounds belonging to each MeSH class were subjected is provided under ‘Task Subgroups’. The 3-task subgroup means drug compounds belonging to the first three MeSH classes presented in the table were used in the single label classification task. Similarly, drugs belonging to the first five MeSH classes were used in the five-task subgroup single label classification task. In addition, all drugs were used in the 12-task subgroup single label classification. The integer values appearing under ‘Label’ are used to represent each MeSH class during modeling for single label tasks.

MeSH Class	Label	Training Samples	Test Samples *	Task Subgroups
Central Nervous System	0	1139	176 (0.13)	3, 5, 12
Antineoplastic	1	1177	347 (0.23)	3, 5, 12
Cardiovascular	2	788	152 (0.16)	3, 5, 12
Gastrointestinal	3	258	46 (0.15)	5, 12
Anti-infective	4	2398	776 (0.24)	5, 12
Reproductive Control	5	148	33 (0.18)	12
Lipid Regulating	6	164	21 (0.11)	12
Hematologic	7	267	47 (0.15)	12
Respiratory System ^†^	8	101	8 (0.07)	12
Anti-inflammatory	9	373	64 (0.15)	12
Urological	10	26	10 (0.28)	12
Dermatological	11	115	18 (0.14)	12

* The value in parentheses represents the proportion of the test drug compounds over the total number of drug compounds in that MeSH class, i.e., $\frac{x}{x+y}$ where *x* is the number of drugs in the test set and *y* is the number of drugs in the training set. ${}^{†}$ Respiratory system agents presented the least proportion of drugs used in the test set, about 7% only.

**Table 2:** Hyperparameters for models used in our experiments.

	Setting for Model for Each of the Task Subgroups			
Hyperparameter	3	5	12	m
Learning rate	0.0005	0.0005	0.0005	0.0005
Batch size	256	256	512	512
Optimizer	Adam	Adam	Adam	Adam
GCN layers	3	3	3	3
Dense layers	2	2	2	2
Dropout	0.4	0.4	0.25	0.25

**Table 3:** Evaluation metrics showing results of the models’ performance on 3-, 5-, and 12-task subgroups. We first compare IMG + CNN and MFP + RF with our GCN model on cross validation. This information is provided in the columns IMG + CNN Val, MFP + RF Val, and GCN Val respectively. In the GCN Test column, we provide results of our GCN model on test data.

Task Subgroup	Metric	IMG + CNN Val	MFP + RF Val	GCN Val	GCN Test
3	Accuracy	0.884±0.0108	0.882±0.0142	0.9003±0.0068	0.8806±0.0099
	BAC	0.879±0.0143	0.870±0.0162	0.8958±0.0076	0.8790±0.0107
	MCC	0.823±0.0168	0.822±0.0217	0.8479±0.0099	0.8122±0.0154
	AUROC	0.970±0.0063	0.978±0.00382	0.9804±0.0029	0.9634±0.0019
	AP	0.950±0.0108	0.978±0.00382	0.9674±0.0050	0.9372±0.0053
5	Accuracy	0.863±0.0104	0.871±0.0070	0.8861±0.0059	0.8835±0.0028
	BAC	0.828±0.0167	0.822±0.0183	0.8478±0.0093	0.8329±0.0103
	MCC	0.811±0.0140	0.821±0.00969	0.8411±0.0081	0.8220±0.0047
	AUROC	0.972±0.0046	0.981±0.00284	0.9820±0.0020	0.9624±0.0035
	AP	0.933±0.0093	0.950±0.00582	0.9534±0.0042	0.9092±0.0084
12	Accuracy	0.834±0.0084	0.838±0.00677	0.8627±0.0085	0.8676±0.0035
	BAC	0.735±0.0258	0.719±0.02480	0.7734±0.0333	0.7988±0.0066
	MCC	0.793±0.0105	0.797±0.00831	0.8290±0.0108	0.8173±0.0047
	AUROC	0.969±0.0026	0.977±0.00227	0.9836±0.0026	0.9695±0.0014
	AP	0.900±0.0073	0.918±0.00392	0.9269±0.0088	0.8760±0.0041

BAC is balanced accuracy; AP is average precision; MCC is Matthew’s correlation coefficient; AUCROC is Area under ROC curve.

**Table 4:** Results of multi-label classification for the 8336 molecules with an average of 1.2 classes assigned to each molecule for validation and 4610 molecules with an average of 2.7 classes assigned to each molecule for testing. We first compare the previous IMG + CNN to our GCN model on cross validation. This information is provided in the columns IMG + CNN Val and GCN Val, respectively. In addition, in the GCN Test column, we provide results of our GCN model on test data. For the $F_{\beta }$ score, β=2 was used, as in previous methods. Notice that this favors recall over precision according to Equation (7).

Task Subgroup	Metric	IMG + CNN Val	GCN Val	GCN Test
m	Accuracy *	0.9540±0.00133	0.9695±0.0023	0.9752±0.0064
( multi-label)	$F_{\beta }$	0.6350±0.01680	0.7971±0.0212	0.8665±0.0460
	AUROC	0.9380±0.00353	0.9580±0.0046	0.9733±0.0039
	AP	0.8370±0.00953	0.8805±0.0070	0.9306±0.0052

$F_{\beta }$ is Fbeta score; AUCROC is Area under ROC curve; AP is average precision; ${}^{*}$ Class score thresholds set to 0.5; Weighted average was used for AUROC and AP.

**Table 5:** This table contains a list of the predicted drug compounds that have been validated by information in the literature. The ‘True MeSH Class’ column provides the list of MeSH classes to which the drug compound belongs, as given in the PubChem data. On the other hand, the ‘Predicted MeSH Class’ column provides the list of MeSH classes our model predicted for each drug compound. The literature that concurs with these predictions is provided in the ‘Evidence of Prediction’ column.

Compound	True MeSH Class	Predicted MeSH Class	Evidence of Prediction
Ginsenoside Rb2	antineoplastic	anti-infective	[30,31,32,33]
	lipid regulating	CNS	
Balofloxacin	anti-infective	urological	[34]
	antineoplastic	antineoplastic	
Dipyridamole	cardiovascular	antineoplastic	[35,36,37]
	hematologic	lipid regulating	
Hypericin	anti-infective	anti-infective	[38,39,40]
	antineoplastic	antineoplastic	
	CNS	urological	
Lacosamide	cardiovascular	antineoplastic	[41,42,43]
	CNS	CNS	
Otilonium bromide	cardiovascular	anti-infective	[44]
	gastrointestinal	urological	
Palmitoylethanolamide	anti-infective	anti-infective	[45,46]
	anti-inflammatory	cardiovascular	
	CNS	urological	
Peppermint oil	CNS	anti-infective	[47,48]
	gastrointestinal	cardiovascular	
Tirofiban	cardiovascular	CNS	[49,50,51]
	hematologic	antineoplastic	

## Data Availability

All the source code and data are available as a bitbucket repository at https://bitbucket.org/mapogota/medical-subheading/src/master/.

## Supplementary Materials

The following are available online at https://www.mdpi.com/article/10.3390/pharmaceutics13111906/s1, Figure S1. A bar plot showing the number of drugs count on each edge type. These numbers form the weights of the networks of Figure 7 in the manuscript. The exact numbers are provided in Table S1 of this supplementary file. Table S1. Table showing the number of true and predicted drugs that belong to each edge type. Each edge type connects two MeSH classes to which the given and predicted number of drugs belong.

## Abbreviations

The following abbreviations are used in this manuscript:

MeSH	Medical SubHeading
GCN	Graph Convolutional Networks
CNN	Convolutional Neural Networks
UMAP	Uniform Manifold Approximation and Projection
RF	Random Forest
ReLU	Rectified Linear Unit
FC	Fully Connected
SMILES	Simplified Molecular Input Line Entry System
CNS	Central Nervous System
siRNA	Small interfering RNA
ALS	Amyotrophic Lateral Sclerosis
PEA	PalmitoylEthanolAmide
DIP	Dipyridamole
UTI	Urinary Tract Infection
HMGCS1	3-hydroxy-3-methylglutaryl-CoA synthase 1
SOD1	Cu,Zn-superoxide dismutase gene
API	Application Programming Interface
