# Supplementary material for: Drug Therapeutic-Use Class Prediction and Repurposing Using Graph Convolutional Networks
Source: Pharmaceutics. 2021 Nov 10;13(11):1906. doi: 10.3390/pharmaceutics13111906 (PMC8622176; doi:10.3390/pharmaceutics13111906)
Supplement: Supplementary file 1 [file pharmaceutics-13-01906-s001.zip › pharmaceutics-1419008-supplementary.pdf]

pharmaceutics

Supplementary Materials: Drug Therapeutic-Use Class Prediction and Repurposing Using Graph Convolutional Networks

Mapopa Chipofya, Hilal Tayara and Kil To Chong

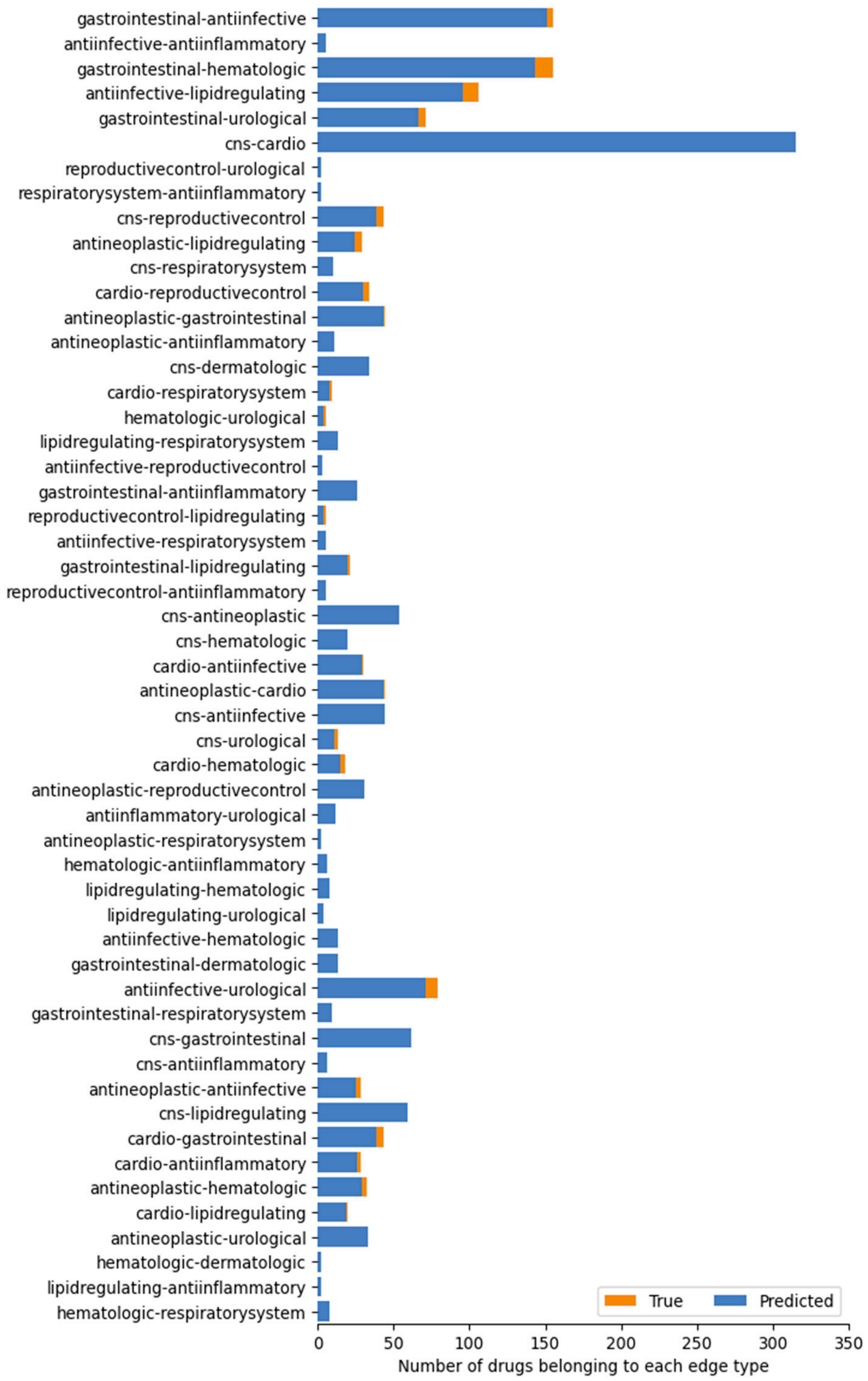

Figure S1. A bar plot showing the number of drugs count on each edge type. These numbers form the weights of the networks of Figure 7 in the manuscript. The exact numbers are provided in Table S1 of this supplementary file.

Table S1. Table showing the number of true and predicted drugs that belong to each edge type. Each edge type connects two MeSH classes to which the given and predicted number of drugs belong.

Edge Type	Number of True Edges	Number of Predicted Edges
gastrointestinal-antiinfective	155	151
antiinfective-antiinflammatory	5	5
gastrointestinal-hematologic	155	143
antiinfective-lipidregulating	106	96
gastrointestinal-urological	71	66
cns-cardio	315	315
reproductivecontrol-urological	2	2
respiratorysystem-antiinflammatory	1	2
cns-reproductivecontrol	43	39
antineoplastic-lipidregulating	29	24
cns-respiratorysystem	8	10
cardio-reproductivecontrol	34	30
antineoplastic-gastrointestinal	44	43
antineoplastic-antiinflammatory	11	11
cns-dermatologic	6	34
cardio-respiratorysystem	9	8
hematologic-urological	5	4
lipidregulating-respiratorysystem	13	13
antiinfective-reproductivecontrol	3	3
gastrointestinal-antiinflammatory	26	26
reproductivecontrol-lipidregulating	5	4
antiinfective-respiratorysystem	4	5
gastrointestinal-lipidregulating	21	20
reproductivecontrol-antiinflammatory	5	5
cns-antineoplastic	50	54
cns-hematologic	18	20
cardio-antiinfective	30	29
antineoplastic-cardio	44	43
cns-antiinfective	40	44
cns-urological	13	11
cardio-hematologic	18	15
antineoplastic-reproductivecontrol	31	31
antiinflammatory-urological	12	12
antineoplastic-respiratorysystem	1	2
hematologic-antiinflammatory	5	6
lipidregulating-hematologic	7	8
lipidregulating-urological	4	4
antiinfective-hematologic	11	13
gastrointestinal-dermatologic	11	13
antiinfective-urological	79	71
gastrointestinal-respiratorysystem	9	9
cns-gastrointestinal	55	62
cns-antiinflammatory	6	6
antineoplastic-antiinfective	28	25
cns-lipidregulating	57	59
cardio-gastrointestinal	43	39
cardio-antiinflammatory	28	26
antineoplastic-hematologic	32	29

cardio-lipidregulating	20	19
antineoplastic-urological	33	33
hematologic-dermatologic	1	2
lipidregulating-antiinflammatory	2	2
hematologic-respiratorysystem	8	8
